# Mechanochemical Synthesis of Pt/Nb_2_CT_x_ MXene Composites for Enhanced Electrocatalytic Hydrogen Evolution

**DOI:** 10.3390/ma14092426

**Published:** 2021-05-06

**Authors:** Xiaoyuan Fan, Peng Du, Xiaoxuan Ma, Ruyue Wang, Jingteng Ma, Yonggang Wang, Dongyu Fan, Yuanzheng Long, Bohan Deng, Kai Huang, Hui Wu

**Affiliations:** 1 State Key Laboratory of Information Photonics and Optical Communications, School of Science, Beijing University of Posts and Telecommunications, Beijing 100876, China; fxy2018212527@bupt.edu.cn (X.F.); pengdu@bupt.edu.cn (P.D.); kristenmxx@bupt.edu.cn (X.M.); Wang_Ruyue@bupt.edu.cn (R.W.); mjt2021@bupt.edu.cn (J.M.); wangyg@bupt.edu.cn (Y.W.); vandy@bupt.edu.cn (D.F.); 2 State Key Laboratory of New Ceramics and Fine Processing, School of Materials, Science and Engineering, Tsinghua University, Beijing 100084, China; longyz19@mails.tsinghua.edu.cn (Y.L.); dbh20@mails.tsinghua.edu.cn (B.D.)

**Keywords:** hydrogen evolution, Pt-Nb alloy, mechanochemical synthesis, thermal annealing

## Abstract

Production of hydrogen from water splitting has been considered as a promising solution for energy conversion and storage. Since a noble metal-based structure is still the most satisfactory but scarce kind of catalyst, it is significant to allow for practical application of such catalysts by engineering the heterogeneous structure and developing green and facile synthetic strategies. Herein, we report a mechanochemical ball milling synthesis of platinum nanoclusters immobilized on a 2D transition metal carbide MXene (Nb_2_CT_x_) as an enhanced catalyst for hydrogen evolution. After annealing at 600 °C, ultrafine Pt_3_Nb nanoclusters are formed on the Pt/Nb_2_CT_x_ catalyst. As prepared, the Pt/Nb_2_CT_x_-600 catalyst demonstrates superior electrochemical HER activity and stability with an ultralow overpotential of 5 mV and 46 mV to achieve 10 mA cm^−2^ and 100 mA cm^−2^, respectively, in comparison with other Nb_2_CT_x_-based catalysts and commercial Pt/C catalysts. Moreover, the remarkable durability is also confirmed by accelerated durability tests (ADTs) and long-term chronoamperometry (CA) tests. The excellent HER performance was attributed to high Pt dispersion and more active site exposure by the mechanochemical process and thermal treatment. Such results suggest that the mechanochemical strategy provides a novel approach for rational design and cost-effective production of electrocatalysts, also providing other potential applications in a wide range of areas.

## 1. Introduction

With the development of modern industry, a rapidly increasing demand for energy has become one of the most significant problems affecting the life of human beings [1,2]. Hydrogen has received substantial attention as a renewable and completely clean energy carrier, making it a favorable candidate for replacing traditional fossil fuels and a promising solution for alleviating environmental crisis [3,4,5]. The production of hydrogen from water splitting represents a highly efficient and sustainable approach to deal with this concern. Thus, novel hydrogen evolution reaction (HER) electro-catalysts exhibiting excellent activity and long durability, along with a green and scalable synthesis strategy, are highly desired for the realization of clean energy infrastructure and application [6,7].

In the past few years, state-of-the-art noble metal-free catalysts with high HER activity have been extensively reported, consisting of carbon-based nanostructures, metal phosphides, MoS_2_, transition metal oxides and related compounds [8,9,10,11,12]. Nevertheless, noble metal platinum-based catalysts are still the most promising HER electrocatalysts currently, due to their advantageous catalytic activity and selectivity, but the extremely high costs, difficulty of engineering and mass-producing desirable structures with high performance seriously obstruct industrial application [13]. As we know, electrocatalytic reaction occurs on the surface and interface of the catalyst, while in the process of preparation and long-term working noble metal nanoclusters or particles supported on substrate materials are prone to agglomeration, or active sites are blocked due to high surface free energy, which causes the active noble metal component to exist in the form of larger aggregates, leading to loss of catalytic activity [14,15,16]. In addition, the preparation of such supported noble metal catalysts generally requires complicated procedures, complicated experimental conditions (such as high annealing temperature and pressure) and the use of a large number of organic solvents or capping agents, making the processing inefficient in time and costs, and even bringing environmental problems [17,18]. Moreover, it is a challenge to apply synthetic routes from a laboratory scenario to large-scale industrial production, since the scale-up effect will make it difficult to delicately control the size and dispersity of noble metal nanostructures on supports [19]. The problems mentioned above have appreciably limited the large-scale application of such catalysts in practical scenarios. Therefore, it is urgent to design a geometric and electronic structure for supported noble metal catalysts and develop an economical and facile synthetic strategy for catalysts.

As types of newly emerging 2D material, MXenes, early transition-metal carbides (M) and carbonitrides (M) have a well-defined structure and a wide range of adjustable components and have received substantial attention. The general formula of MXene is M_n+1_X_n_T_x_ (n = 2–4); the layers containing the transition metals are exposed outside of the structure and T_x_ generally stands for surface termination groups containing OH*, O* and F* [20,21]. The excellent electronic conductivity, volumetric capacitance, and chemical stability of MXene make it favorable as a promising support material for metal nanostructures and it is widely used in energy conversion and storage, sensing, biomedicine and other fields [22,23,24]. The mechanical exfoliation and downsizing of layered MXene at room temperature can significantly expose more electrochemical active sites and increase surface-to-volume ratio. At the same time, the noble metal nanostructures are compounded, which can optimize the dispersion of noble metal species and the formation of a metal-support interface with high thermal stability [25].

The existence of noble metal nanostructures (single atoms, clusters or nanoparticles) not only promotes the removal of surface functional groups but also accelerates the electron transfer between MXene support and Pt nanoclusters [26]. Furthermore, metal-support interactions (MSIs) can become enhanced through thermal annealing under inert conditions at a moderate temperature, meanwhile generating a Pt-M (early transition metal) alloy composition and preventing aggregation of the noble metal structure [27,28]. Therefore, MXene was adopted as substrate material and simultaneously achieved the exfoliation of 2D layers of a smaller size, as well as immobilization of noble metal nanostructures by a mechanochemical process, providing an optimized interface area and improvement of MSIs, which paves the way for superior HER performance.

In this study, we developed a mechanochemical ball milling synthesis to fabricate a Pt-based Nb_2_CT_x_ MXene composite, the Pt loading amount of which was 1.0 wt%. Subsequently thermal treatment at 600 °C guarantees the rearrangement of atoms and electrons on the surface of the Pt and Nb_2_CT_x_ substrate, forming a small-sized Pt_3_Nb alloy with an average size of only 2.1 nm. Electrochemical tests showed that the catalyst possessed superior HER performance and long-term stability in acidic electrolyte. This work provides a simple and universal mechanochemical strategy for the large-scale preparation of transition metal-Pt alloy supported MXene composite catalyst with a wide range of compositions and structures, which also expands the practical application of such catalysts in diverse fields.

## 2. Materials and Methods

### 2.1. Materials

Nb_2_CT_x_ (55–70 wt%, XFNANO, Nanjing, China), Chloroplatinic acid (H_2_PtCl_6_·6H_2_O, AR, Pt ≥ 37.5%, Aladdin, Shanghai, China), and absolute ethanol (C_2_H_5_OH, 99.8%, Aladdin, Shanghai, China) were used as received without any further purification.

### 2.2. Preparation of Nb_2_CT_x_ Based Catalysts

Typically, for the ball milling synthesis of Pt/Nb_2_CT_x_, 500 mg of bulk Nb_2_CT_x_ powder was immersed into 30 cm^3^ of absolute ethanol and sonicated by a high-power ultrasonic probe for 30 min to form a homogenous suspension. Then 420 uL of 0.1 mol/L solution of chloroplatinic acid was introduced into as-prepared suspension and continuously stirred for 40 min. The mixed solution was then transferred into a corundum tank and ball-milled at a speed of 150 rpm for about 30 min. Finally, the Pt/Nb_2_CT_x_ sample was obtained by vacuum filtration and then drying the solution. The as-prepared Pt/Nb_2_CT_x_ catalyst was annealed at 600 °C for 2 h with a heating rate of 1 °C min^−1^ under flowing Ar gas to obtain the sample denoted as Pt/Nb_2_CT_x_-600.

### 2.3. Characterizations of Nb_2_CT_x_ Based Catalysts

The morphology, microstructure and chemical composition of the as-prepared catalysts were characterized by energy dispersion spectroscopy (EDS) elemental mapping images taken by aberration-corrected high-resolution transmission electron microscopy (HRTEM, JEOL, Tokyo, Japan, JEM-ARM200F), as well as a field emission scanning electron microscope (FE-SEM, LEO-1530, Zeiss, Oberkochen, Germany). The structural characterization of catalysts was performed by powder X-ray diffractometry (XRD, RIGAKU, Tokyo, Japan, D/max 2500 V) using Cu Ka as radiation source, and X-ray photoelectron spectroscopy (XPS, ESCALAB 250Xi, Thermo Scientific, Waltham, MA, USA). Inductively coupled plasma atomic emission spectrometry (ICP-AES) analysis was performed on a Thermo ICAP-6300 instrument (Thermo Electron Limited, Cambridge, UK). The electrocatalytic measurements of Nb_2_CT_x_ based catalysts are shown in the Supplementary Materials.

## 3. Results

**Figure 1:**
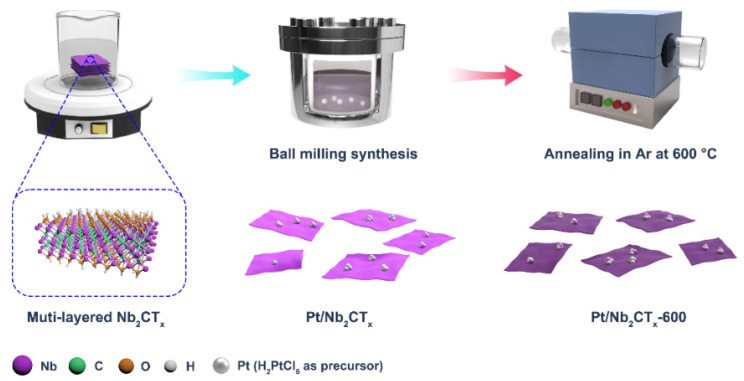
Schematic illustration of the preparation ofNb_2_CT_x_-600 catalyst by ball milling and thermal treatment.

**Figure 2:**
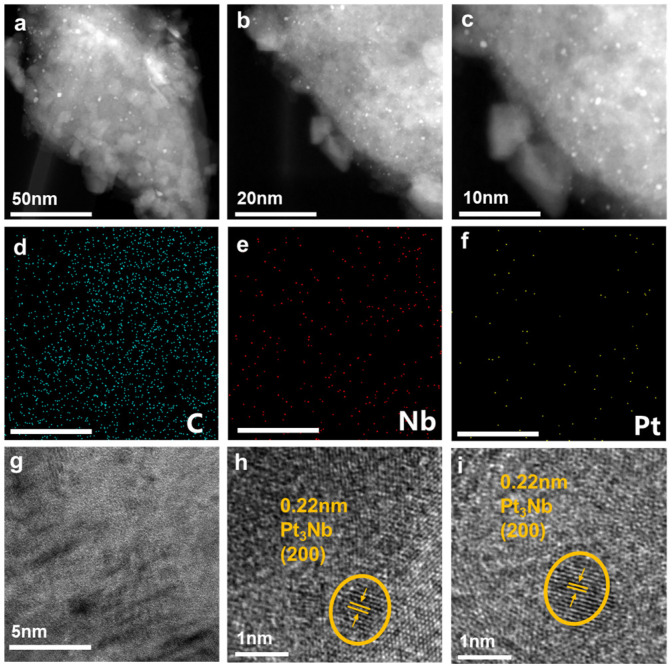
(**a**–**c**) TEM images of Pt/Nb_2_CT_x_-600; (**d**–**f**) EDS mapping images of Pt/Nb_2_CT_x_-600 (scale bar: 50 nm); (**g**–**i**) HRTEM of Pt/Nb_2_CT_x_-600.

The synthetic procedure for Pt/Nb_2_CT_x_ including ball milling and thermal treatment is illustrated in Figure 1. We employed ethanol with slow reduction kinetics to guarantee that the H_2_PtCl_6_ precursor was chemically reduced into relatively small sized nanostructures and adequately combined with the Nb_2_CT_x_ substrate [29]. The solution of H_2_PtCl_6_ and Nb_2_CT_x_ was directly transferred into a corundum tank for further ball milling at room temperature. As shown in Figure S1, the SEM images of Pt/Nb_2_CT_x_ after the ball milling processes indicate that the bulky layered Nb_2_CT_x_ MXene materials can be partly exfoliated into an island structure with an average size of about 500 nm, which is closely correlated with the fully exposure of active sites. Moreover, the appropriate mechanical force in the process plays an important role in controlling the Pt nanocluster’s size and distribution [30]. In the next step, as-prepared Pt/Nb_2_CT_x_ was annealed in Ar atmosphere at 600 °C (designated as Pt/Nb_2_CT_x_-600) to obtain thermally stable Pt-Nb alloys and optimize the electrochemical HER performance. The morphology of Pt and Nb_2_CT_x_ compounds was firstly investigated by transmission electron microscopy (TEM). The TEM images of Pt/Nb_2_CT_x_-600 catalyst at different magnifications are shown in Figure 2a–c, and metallic nanoclusters can be observed, well dispersed onto the surface of Nb_2_CT_x_ substrate, the average diameter of which is about 1.5 nm. As shown in Figure S2, we also observed metallic nanoclusters of similar size immobilized on the surface of substrate materials for the Pt/Nb_2_CT_x_ catalyst, indicating that Pt species might react with ethanol and anchor onto Nb_2_CT_x_ during the mechanochemical ball milling procedure. Moreover, the homogeneous distribution of Pt nanoclusters was also revealed by the energy dispersive spectroscopy (EDS) elemental mapping of Nb, C and Pt elements in Figure 2d–f. The loading amount of Pt in the catalyst is as low as 1.0 wt%, which is determined by ICP-AES. As shown in Figure 2g–i, high-resolution transmission electron microscopy (HRTEM) further proves that the existence of Pt_3_Nb alloy nanoclusters successfully anchored on Nb_2_CT_x_ with a typical size of about 1.2 nm and a representative crystal lattice indexed to (200) planes (0.22 nm in space) of Pt_3_Nb alloy, which matches the schematic illustration and material design [31].

As shown in Figure S3, selective area electron diffraction (SAED) was carried out to obtain more accurate information on the lattice, where both pristine Nb_2_CT_x_ and Pt/Nb_2_CT_x_-600 samples show good crystallinity of the Nb_2_CT_x_ ultrathin nanostructures. In addition, Figure S4 displays the intensity profile across the lattice of pristine Nb_2_CT_x_ and Pt/ Nb_2_CT_x_, the similar grayscale value suggesting that the crystalline structure of the Pt/Nb_2_CT_x_-600 has not changed, while the appearance of peak at 2520 and 3112 μm^−1^ can be attributed to the formation of Pt_3_Nb and the enhanced metal-support interaction.

**Figure 3:**
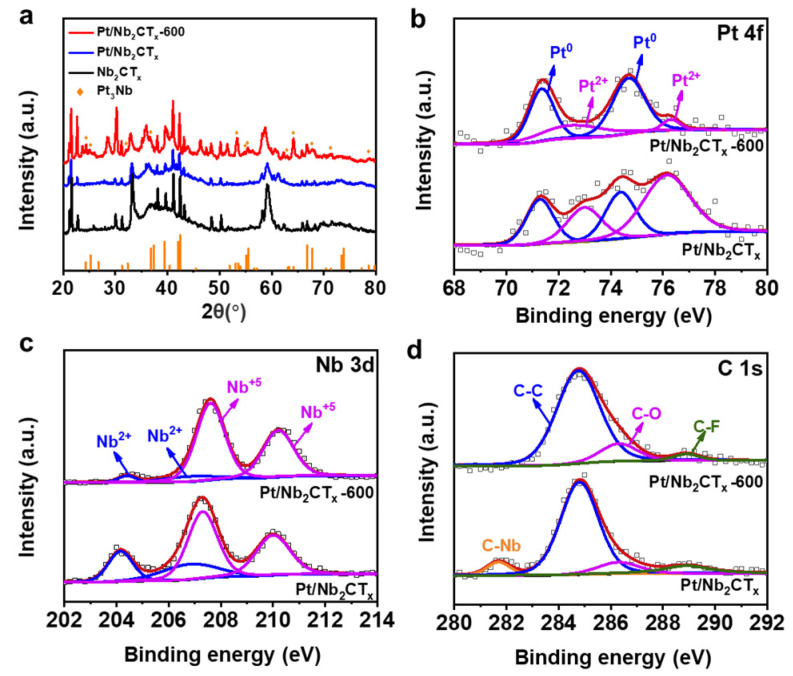
(**a**) XRD patterns of bulk Nb_2_CT_x_, Pt/Nb_2_CT_x_, Pt/Nb_2_CT_x_-600; (**b**–**d**) Pt 4f, Nb 3d, C 1s XPS spectrum of Pt/Nb_2_CT_x_ and Pt/Nb_2_CT_x_-600 catalysts.

X-ray diffraction (XRD) and X-ray photoelectron spectroscopy (XPS) characterizations were further investigated to obtain insight into the chemical state and coordination environment of as-prepared catalysts. As featured in Figure 3a, due to the abundant surface termination groups T_x_, the diffraction peaks of Nb_2_CT_x_ were only partly consistent with the standard card of Nb_2_C (shown in Figure S5). No obvious Pt phase could be identified in the XRD pattern of Pt/Nb_2_CT_x_, suggesting the homogeneous distribution of small-sized Pt nanoclusters, and no obvious changes were induced to the crystalline structure of Nb_2_CT_x_ by the Pt modification. In comparison, the annealed Pt/Nb_2_CT_x_-600 sample exhibits a dramatic appearance of characteristic peaks corresponding to the standard card (JCPDS, PDF#78-0499) for Pt_3_Nb alloy, the relatively weak signal of which can be attributed to the low Pt modification content [32]. This result confirms that Pt_3_Nb alloy was generated due to annealing treatment in Ar at 600 °C, consistent with the HRTEM and SAED results. Figure S6 exhibits the whole spectrum survey of Pt/Nb_2_CT_x_ and Pt/Nb_2_CT_x_-600 calibrated with the main peak of C 1s at 284.8 eV, indicating the existence of Nb, C, O, and F elements. In Figure 3b, the high-resolution Pt 4f XPS spectrum of Pt/Nb_2_CT_x_ and the annealed catalyst are resolved into four peaks corresponding to Pt ^0^ in a metallic state existing on the surface of Nb_2_CT_x_ along with some Pt^2+^ (Pt-O) species [33,34]. Compared with the Pt 4f _7/2_ spectrum of Pt/Nb_2_CT_x_ catalyst, a shift toward higher binding energy (0.2~0.4 eV for Pt ^0^ and 0.1 eV for Pt^2+^) can be notably observed for the annealed sample. The dominant existence of oxidation states of niobium can be brought out according to the Nb 3d spectrum in Figure 3c, which is due to the oxophilicity of Nb. For the unannealed catalyst, the Nb 3d XPS peak can be divided into two group components corresponding to Nb^2+^ (204.1, 207 eV) and Nb^5+^ (207.2, 210 eV), respectively. A positive shift (204.6/207.2 eV for Nb ^2+^ and 207.6/210.2 eV for Nb^5+^) can be detected after thermal treatment [35]. The above analysis of Pt 4f and Nb 3d XPS spectrum provides cogent evidence for the amplified metal-support interaction and formation of Pt-Nb alloy. In addition, the C 1s spectrum of Pt/Nb_2_CT_x_ in Figure 3d is deconvoluted into four peaks attributing to C-Nb (281.6 eV), C-C (284.8 eV), C-O (286.2 eV) and C-F (288.8 eV). For the Pt/Nb_2_CT_x_-600, the near disappearance of the C-Nb typical peak can be markedly observed, suggesting the transfer of atoms and electrons between Nb_2_CT_x_ substrate and Pt species under the thermal effect. The surface chemical states of C element also indicate the existence of surface termination groups before and after annealing [36].

**Figure 4:**
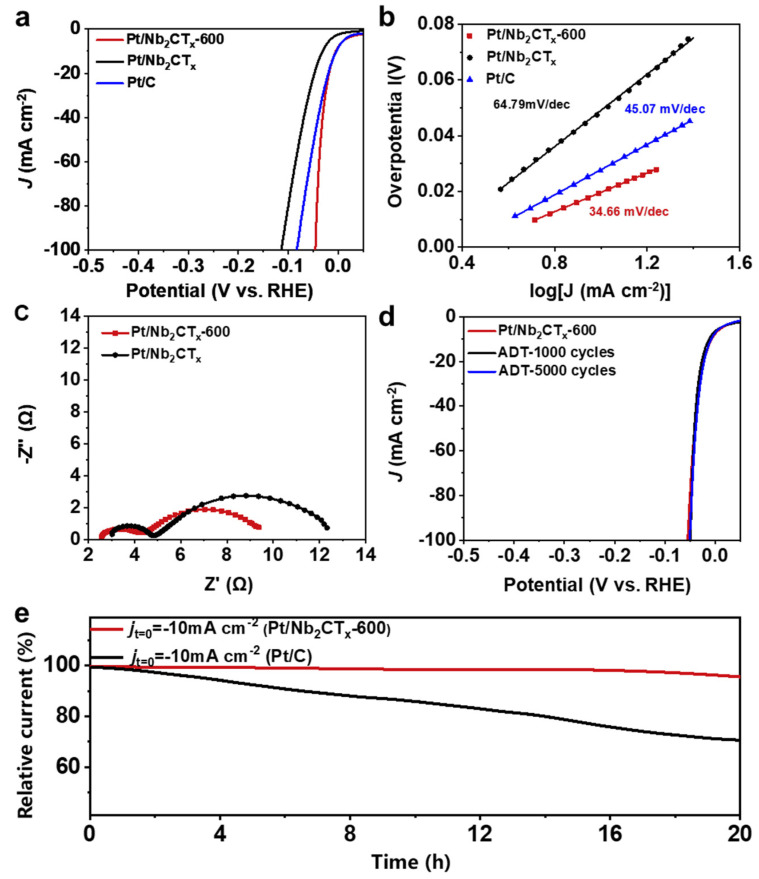
Electrocatalytic HER performance of different catalysts in 0.5 M H_2_SO_4_. (**a**) Polarization curves of the Pt/Nb_2_CT_x_, Pt/Nb_2_CT_X_-600, commercial Pt/C catalysts. (**b**) Tafel plots of the Pt/Nb_2_CT_x_, Pt/Nb_2_CT_X_-600, commercial Pt/C catalysts. (**c**) EIS Nyquist plots for HER performance of the Pt/Nb_2_CT_x_ and Pt/Nb_2_CT_X_-600 catalysts. (**d**) ADT tests for HER performance of Pt/Nb_2_CT_X_-600 catalyst. (**e**) CA curves of Pt/Nb_2_CT_x_ and Pt/C catalysts for HER performance.

The electrocatalytic performance of the Pt/Nb_2_CT_x_-600 and related catalysts for hydrogen evolution reaction (HER) was evaluated using a three-electrode system in the 0.5 M H_2_SO_4_ electrolyte. Linear sweep voltammetry (LSV) is adopted to evaluate the HER activities of the catalysts as shown in Figure 4a. Significantly, the Pt/Nb_2_CT_x_-600 with homogeneous distributed Pt_3_Nb alloys exhibits the superior HER activity compared with Pt/Nb_2_CT_x_, Nb_2_CT_x_ substrate and commercial Pt/C catalysts, requiring an overpotential of only 5 mV and 46 mV to reach a current density of 10 and 100 mA cm^−2^ (*E_j_*_10_ and *E_j_*_100_), respectively, significantly better than the values for Pt/Nb_2_CT_x_ (*E_j_*_10_ = 31 mV, *E_j_*_1__00_ = 115 mV) and commercial Pt/C catalyst (*E_j_*_10_ = 6.2 mV, *E_j_*_1__00_ = 82.4 mV). The results can further be demonstrated by Tafel plots derived from HER polarization curves in Figure 4b. A smaller Tafel slope of 34.66 mV dec^−1^ for Pt/Nb_2_CT_x_-600 than values of 45.07 mV dec^−1^ for Pt/C catalyst and 64.70 mV dec^−1^ for Pt/Nb_2_CT_x_ has been verified following the Volmer-Tafel mechanism, indicating the rapid HER kinetics derived from the advantage of Pt_3_Nb alloy phase. The reaction kinetic rate of catalysts was also evaluated by the electrochemical impedance spectroscopy (EIS) analysis in Figure 4c and Table S1, which is fitted using an equivalent circuit diagram consisting of the solution resistance (*R*_s_), high-frequency interfacial charge transfer resistance (*R*_ct1_) and low-frequency (*R*_2_) element connected with H_2_ mass-transport. The smaller *R*_ct1_ value for the Pt/Nb_2_CT_x_-600 than for the unannealed Pt/Nb_2_CT_x_ delivers an excellent charge-transfer process between Pt-Nb interfaces [37]. In addition, the HER polarization curve and Tafel plot of the pristine Nb_2_CT_x_ substrate are also revealed in Figures S7 and S8, respectively. When the overpotential was up to 500 mV, the pristine Nb_2_CT_x_ catalyst achieved a current density of 36.3 mA cm^−2^ and the corresponding Tafel slope is 239.24 mV dec^−1^, the performance of which is dramatically poorer than Pt immobilized catalysts, indicating that the immobilization of Pt species on the surface of Nb_2_CT_x_ substrate by ball milling synthesis and further thermal treatment can effectively accelerate the charge transfer and kinetics of the catalytic process. Moreover, the excellent catalytic stability of Pt/Nb_2_CT_x_-600 was further convinced using typical accelerated durability tests (ADTs) and long-term chronoamperometry (CA) tests. The obtained polarization curves before and after 1000 and 5000 cycles are shown in Figure 4d, and there is no obvious decay in *E_j_*_10_ and *E_j_*_100_ after 5000 continuous potential cycles. The Pt/Nb_2_CT_x_-600 sample also displays small degradation of about 5% after 20 h CA test operations, compared with ~30% degradation for commercial Pt/C catalysts. As shown in Figures S9–S11, the structural stability of Pt/Nb_2_CT_x_-600 under HER conditions has been confirmed by HRTEM images, XPS and XRD characterizations after a 5k ADT cycles long-term stability test, demonstrating that the active site will not change during the reaction. Above all, the result of the electrochemical test revealed the prominent performance of the Pt/Nb_2_CT_x_-600 catalyst, attributed to the strong MSI between Pt and Nb_2_CT_x_ and the formation of a Pt_3_Nb alloy phase with high intrinsic activity [38]. As discussed above, all electro-catalysis results suggested the advanced HER performance of Pt/Nb_2_CT_x_-600 catalyst via ball milling and thermal treatment. With metal-support interaction, the formation of Pt-Nb alloy could improve the onset potential and benefit the formation of the active site, and the synergetic effect plays its own role in strongly adsorbing H* intermediate species and improves the intrinsic catalytic activity by accelerating kinetics, which will become the key for the synergistically promoted HER. Comparison of *E_j_*_10_, *E_j_*_100_ and Tafel slope with other similar systems is listed in Table S2, confirming that the as-prepared Pt/Nb_2_CT_x_-600 outperformed the majority of other recently reported catalysts.

## 4. Conclusions

In summary, we reported a simple mechanochemical ball milling and annealing strategy for the large-scale synthesis of Pt_3_Nb alloy nanoclusters immobilized on the Nb_2_CT_x_ substrate. The mechanical force and slow chemical kinetics during the process were conductive to the reduction and homogeneous dispersion of Pt species, as well as favoring the mass production of such supported noble metal catalysts. The subsequent thermal treatment in Ar enhanced the metal–support interaction between Pt nanoclusters and Nb_2_CT_x_ substrates, forming Pt_3_Nb alloy, the formation of which was verified by systematic morphology and structural characterization. The electrochemical experiment revealed that the prepared Pt/Nb_2_CT_x_ catalyst exhibits the best HER activity with the lowest overpotential of 5 and 46 mV at the current density of 10 and 100 mA cm^−2^, respectively, better than that of other Nb_2_CT_x_ based catalysts and commercial Pt/C. Long-term stability is also demonstrated over 20 h CA tests and 5000 cycles of ADT tests. The superior performance can be ascribed to the enhanced metal–support interaction as well as the uniformly distributed Pt_3_Nb alloy phase with small size and high intrinsic HER activity. The results suggest that the mechanochemical strategy provides a new approach for green and effective production of supported noble metal catalysts for energy conversion and storage applications.

## Data Availability

The data presented in this study are available on request from the corresponding author.

## Supplementary Materials

The following are available online at https://www.mdpi.com/article/10.3390/ma14092426/s1, Figure S1: Scanning electron microscopic (SEM) images of (a, b) bulk Nb_2_CT_x_; (c, d) Pt/ Nb_2_CT_x_ catalyst synthesized by ball milling, Figure S2: Transmission electron microscopic (TEM) images of Pt/ Nb_2_CT_x_ catalyst synthesized by ball milling, Figure S3: Selective area electron diffraction (SAED) of (**a**) pristine Nb_2_CT_x_ and (**b**) Pt/ Nb_2_CT_x_-600, Figure S4: The intensity profile across the lattice of pristine Nb_2_CT_x_ and Pt/ Nb_2_CT_x_, Figure S5: The XRD pattern of pristine Nb_2_CT_x_, Figure S6: The whole spectrum survey of Pt/Nb_2_CT_x_ and Pt/Nb_2_CT_x_-600 calibrated with the main peak of C 1s at 284.8 eV, Figure S7: The HER polarization curve of the pristine Nb_2_CT_x_ substrate, Figure S8: The HER Tafel plot of the pristine Nb_2_CT_x_ substrate, Figure S9: TEM images of Pt/ Nb_2_CT_x_-600 after a 5k ADT cycles long-term stability test, Figure S10: XRD patterns of Pt/ Nb_2_CT_x_-600 and Pt/ Nb_2_CT_x_-600 after a 5k ADT cycles long-term stability test, Figure S11: Pt 4f XPS spectrum of Pt/ Nb_2_CT_x_-600 after a 5k ADT cycles long-term stability test, Table S1: The EIS fitting results of Pt/Nb_2_CT_x_ and Pt/Nb_2_CT_x_-600 catalysts, Table S2: HER activity for recently reported noble metal catalysts in 0.5 M H_2_SO_4_ (* represents 0.1M H_2_SO_4_). References [39,40,41,42,43,44,45,46,47,48,49,50,51,52,53,54,55,56,57] are cited in the supplementary materials
