# Supplementary material for: Mechanochemical Synthesis of Pt/Nb2CTx MXene Composites for Enhanced Electrocatalytic Hydrogen Evolution
Source: Materials (Basel). 2021 May 6;14(9):2426. doi: 10.3390/ma14092426 (PMC8124981; doi:10.3390/ma14092426)
Supplement: Supplementary file 1 [file materials-14-02426-s001.zip › materials-1183822-supplementary.pdf]

materials

MDPI

Communication

Mechanochemical Synthesis of Pt/Nb_2CT_x MXene Composites for Enhanced Electrocatalytic Hydrogen Evolution

Xiaoyuan Fan [1,†], Peng Du [1,2,†], Xiaoxuan Ma [1,†], Ruyue Wang [1,2], Jingteng Ma [1], Yonggang Wang [1], Dongyu Fan [1], Yuanzheng Long [2], Bohan Deng [2], Kai Huang [1,*] and Hui Wu [2,*]

[1] State Key Laboratory of Information Photonics and Optical Communications and School of Science, Beijing University of Posts and Telecommunications, Beijing 100876, China; fxy2018212527@bupt.edu.cn (X.F.); pengdu@bupt.edu.cn (P.D.); kristenmxx@bupt.edu.cn (X.M.); Wang_Ruyue@bupt.edu.cn (R.W.); mjt2021@bupt.edu.cn (J.M.); wangyg@bupt.edu.cn (Y.W.); vandy@bupt.edu.cn (D.F.); huang-kai@bupt.edu.cn (K.H.)

[2] State Key Laboratory of New Ceramics and Fine Processing, School of Materials, Science and Engineering, Tsinghua University, Beijing 100084, China; longyz19@mails.tsinghua.edu.cn (Y.L.); dbh20@mails.tsinghua.edu.cn (B.D.)

† These authors contribute equally to this work.

* Correspondence: huang-kai@bupt.edu.cn (K.H.); huiwu@mail.tsinghua.edu.cn (H.W.)

Electrocatalytic measurements of Nb_2CT_x based catalysts:

The electrochemical measurements for HER were performed by an Autolab potentiostat (PGSTAT-204N) in a three-electrode system in N_2-saturated 0.5 M H_2SO_4 electrolyte, with a loading of 0.5 mg cm^{-2} for Nb_2CT_x-based catalysts and 1 mg cm^{-2} for 20 wt% commercial Pt/C catalyst. All of the catalysts were dropped onto the carbon paper as the working electrode, the surface area where catalysts were dropped was 0.2 cm^2, followed by drying at room temperature. An SCE (Ag/AgCl with saturated KCl as the filling solution) was used as the reference electrode and graphite rod was used as the counter electrode. All polarization curves were calibrated versus RHE (reversible hydrogen electrode) according to the following equation: $E_{RHE} = E_{Ag/AgCl} + 0.1989 + 0.059 \times pH$, with an IR compensation level of 90%. The electrochemical impedance spectroscopy (EIS) analysis was completed during the frequency between 100 kHz to 0.1 Hz under the potential of −0.2 V with an amplitude of 5 mV in 0.5 M H_2SO_4 solution. The durability test was evaluated by chronoamperometry (CA) measurements at an initial current density of 10 mA cm^{-2} for 24 h as well as accelerated durability test (ADT) using cyclic voltammetry (CV) cycles at a scan rate of 100 mV s^{-1} for 5000 cycles.

Supplementary Figures

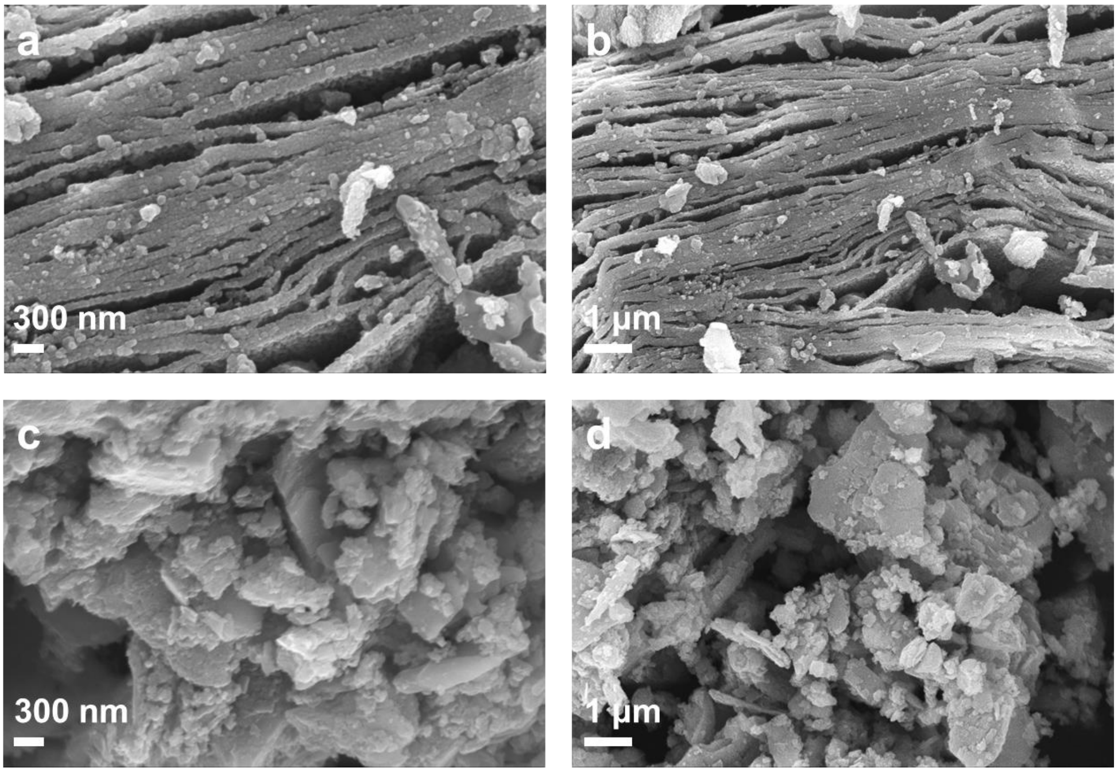

Figure S1. Scanning electron microscopic (SEM) images of (**a**,**b**) bulk Nb_2CT_x; (**c**,**d**) Pt/ Nb_2CT_x catalyst synthesized by ball milling.

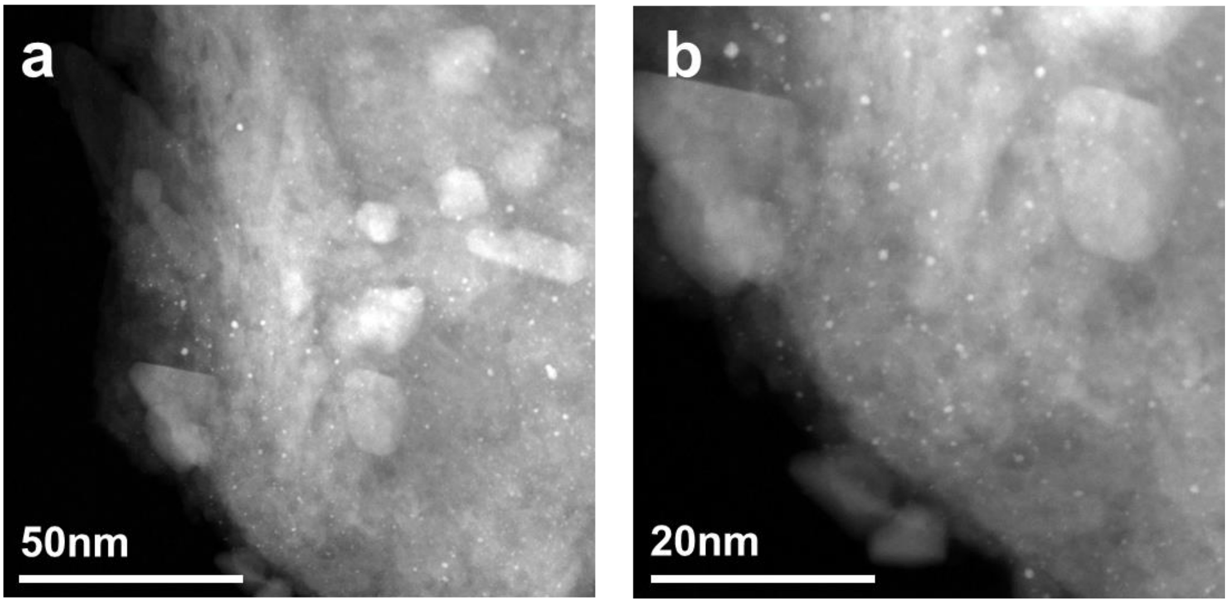

Figure S2. Transmission electron microscopic (TEM) images of Pt/ Nb_2CT_x catalyst synthesized by ball milling, scale bar: 50 nm for (**a**), 20 nm for (**b**).

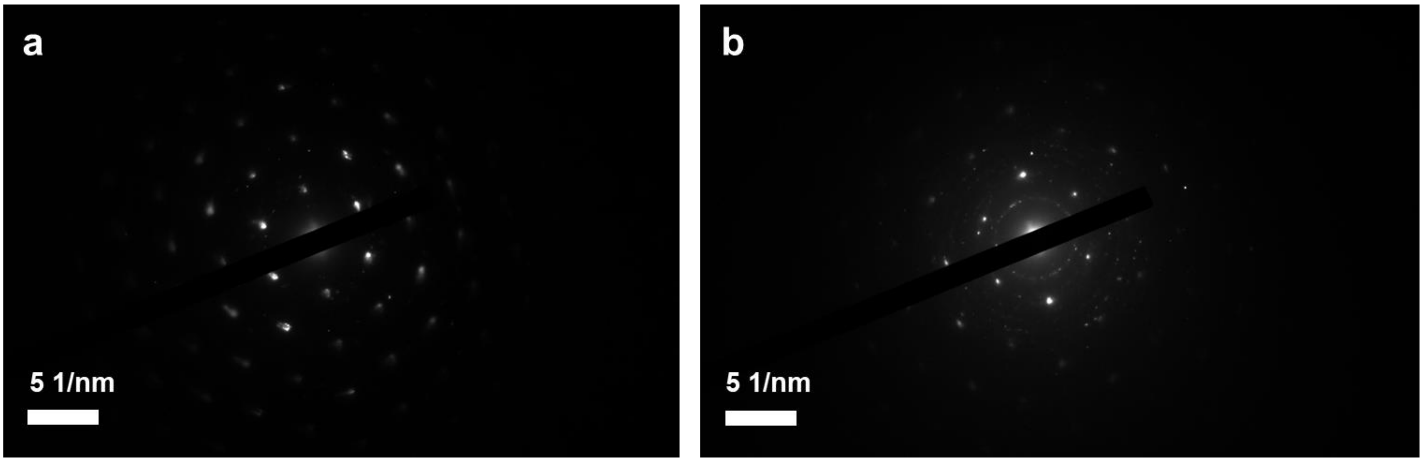

Figure S3. Selective area electron diffraction (SAED) of (**a**) pristine Nb_2CT_x and (**b**) Pt/ Nb_2CT_x-600.

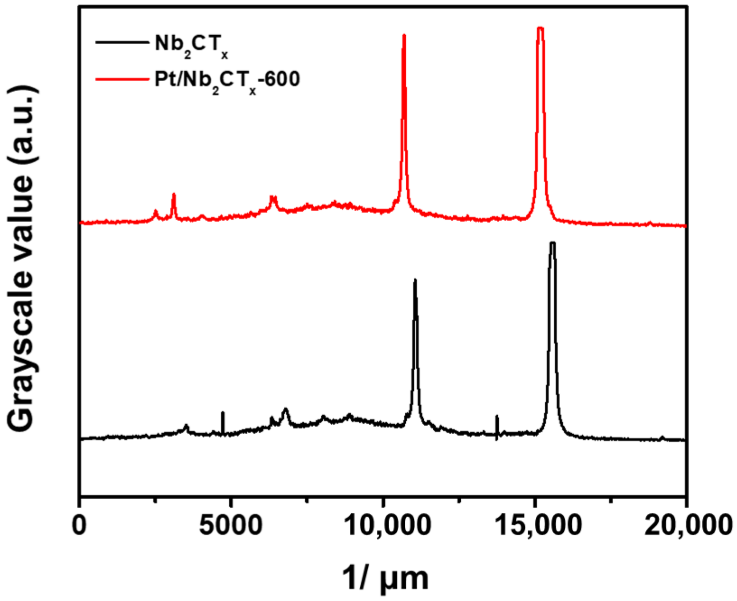

Figure S4. The intensity profile across the lattice of pristine Nb_2CT_x and Pt/ Nb_2CT_x.

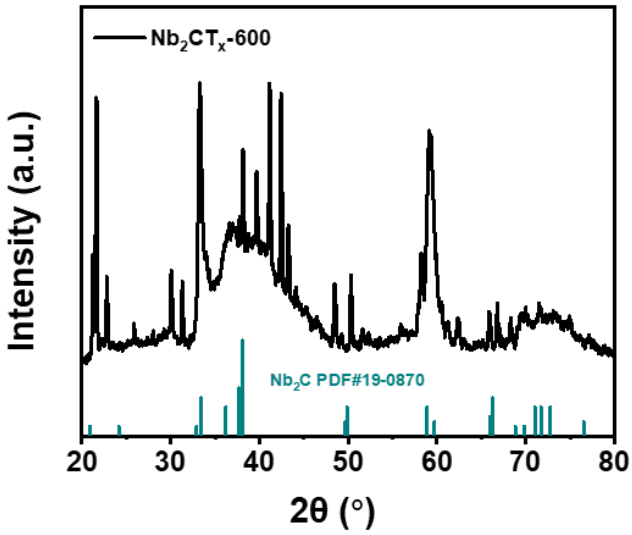

Figure S5. The XRD pattern of pristine Nb_2CT_x.

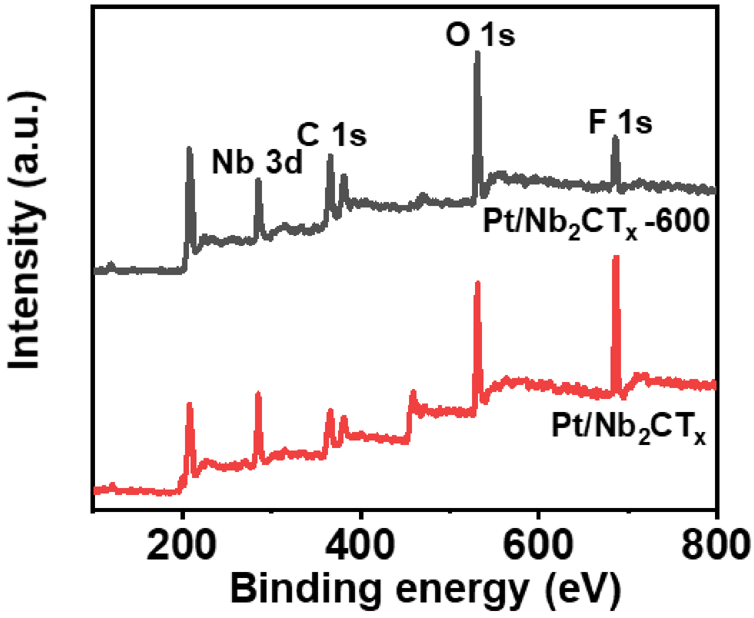

Figure S6. The whole spectrum survey of Pt/Nb_2CT_x and Pt/Nb_2CT_x-600 calibrated with the main peak of C 1s at 284.8 eV

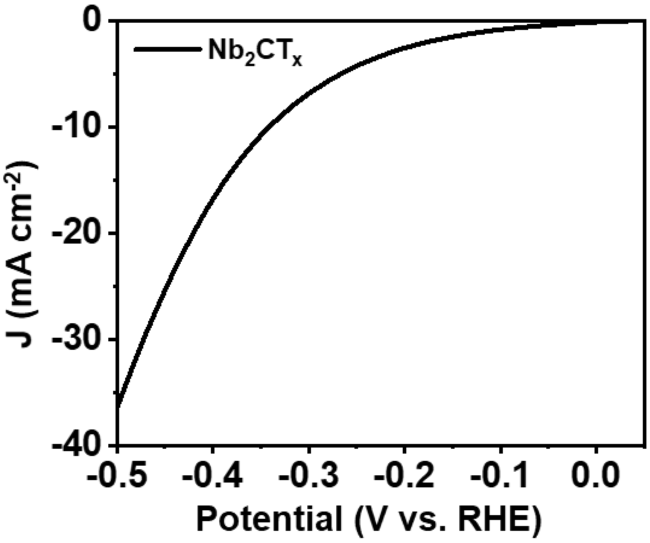

Figure S7. The HER polarization curve of the pristine Nb_2CT_x substrate

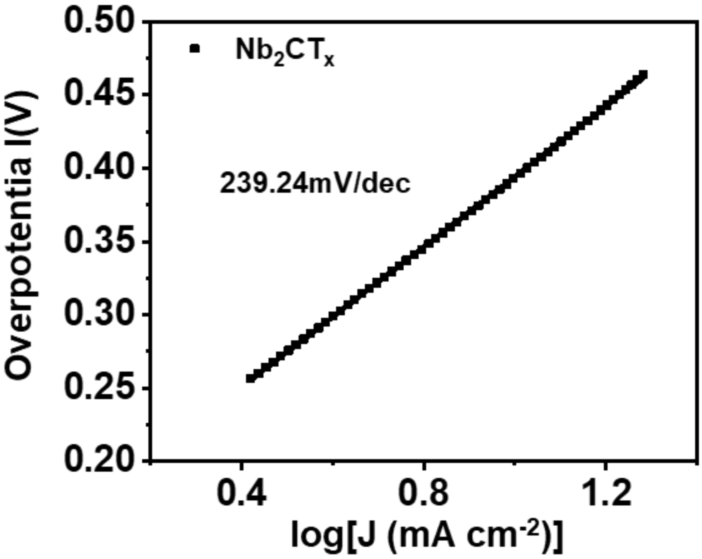

Figure S8. The HER Tafel plot of the pristine Nb_2CT_x substrate.

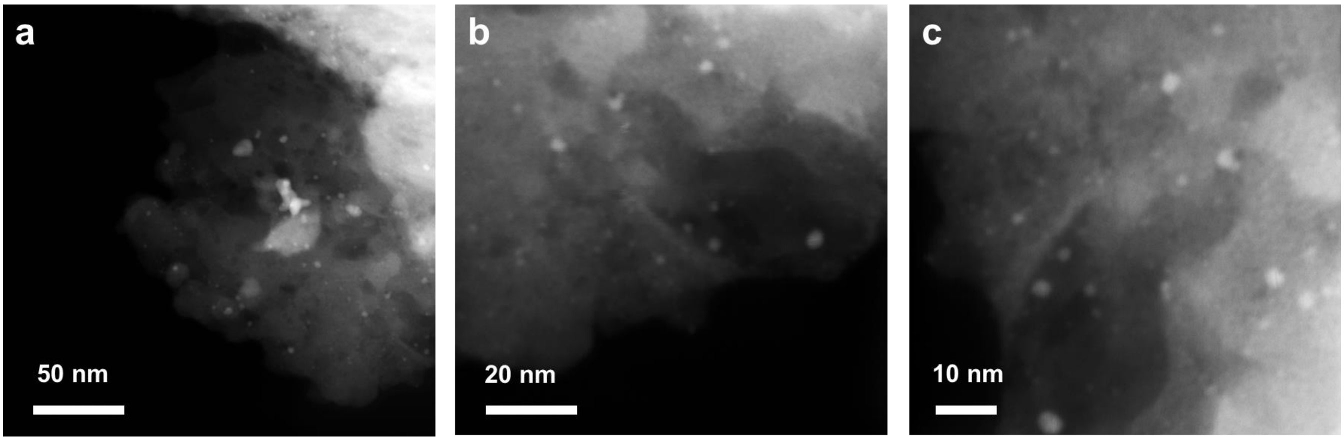

Figure S9. TEM images of Pt/ Nb_2CT_x-600 after a 5k ADT cycles long-term stability test, scale bar: 50 nm for (**a**), 20 nm for (**b**) and 10 nm for (**c**).

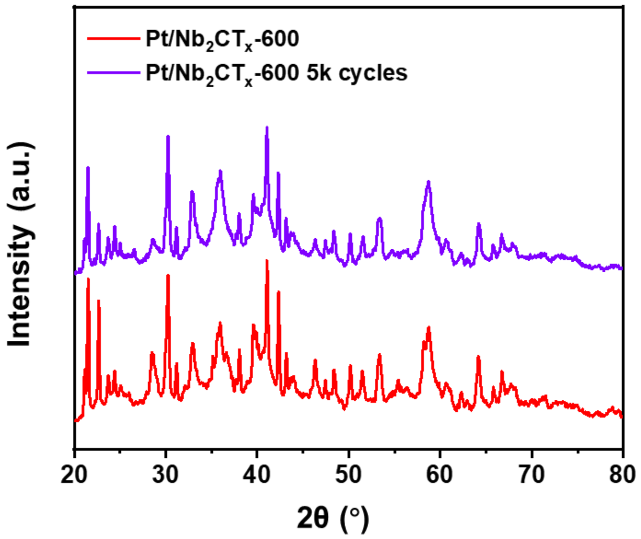

Figure S10. XRD patterns of Pt/ Nb_2CT_x-600 and Pt/ Nb_2CT_x-600 after a 5k ADT cycles long-term stability test.

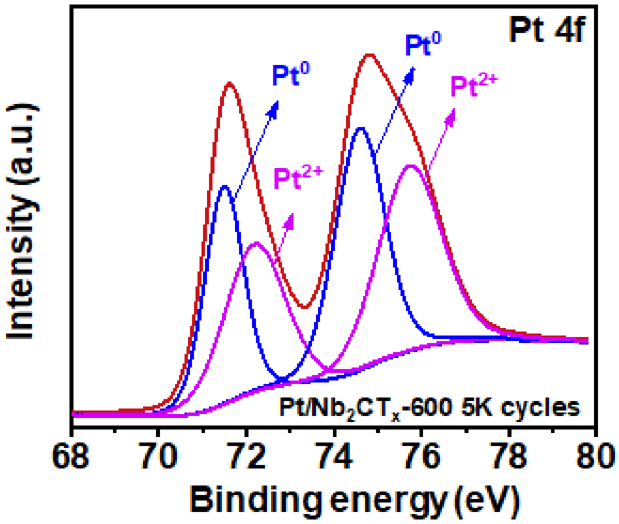

Figure S11. Pt 4f XPS spectrum of Pt/ Nb_2CT_x-600 after a 5k ADT cycles long-term stability test.

Table S1. The EIS fitting results of Pt/Nb_2CT_x and Pt/Nb_2CT_x-600 catalysts. The surface area where catalysts were dropped onto the carbon paper was 0.2 cm^2

Catalysts	R_s (Ω cm^2)	R_{ct1} (Ω cm^2)	R_2 (Ω cm^2)
Pt/Nb_2CT_x	0.494	0.372	1.126
Pt/Nb_2CT_x-600	0.582	0.386	1.642

Table S2. HER activity for recently reported noble metal catalysts in 0.5 M H_2SO_4 (* represents 0.1M H_2SO_4). For the noble metal-based catalysts, the loading was corresponding to the total content of Pt/. For other kinds of catalysts, the loading was corresponding to the whole quality of catalysts.

Catalyst	Pt loading amount (mg cm^{-2})	Tafel slope (mV dec^{-1})	Overpotential at 10 mA cm^{-2} (mV)	Overpotential at 100 mA cm^{-2} (mV)	Ref.
**Pt/Nb_2CT_x**	**0.010**	34.66	5	46	This work
PtW_6O_{24}/C	N/A	29.8	22	65	39
Pt@DNA	0.015	30	21	N/A	40
Pt/f-MWCNTs	0.0012	31	43.9	~83	41
Pt SA/m-WO_{3-x}	N/A	45	38	N/A	42
Pt-WO_3	N/A	32.3	39	N/A	43
Pt/Ti_3C_2-550	0.02	32.3	32.7	N/A	44
Pt–NiFe-LDH	N/A	46	~47	~172	45
$Mo_2TiC_2T_x$–Pt_{SA}	1.0	30	30	77	46
Pt/NMC-LT	0.010	26.3	17.0	49.8	47
Pt1/hNCNC-2.92	0.003	24	15	N/A	48
CS-PdPt	0.26	33	26	N/A	49
Pt@mh-3D	0.2	24.2	13	N/A	22
Mo_2CT_x/2H-MoS_2	N/A	60	119	N/A	50
CoP/Ti_3C_2	N/A	57.6	71	N/A	51
RuSA-N-$Ti_3C_2T_x$	0.015	42	27	N/A	52
Ru-SA/$Ti_3C_2T_x$	N/A	27.7	70	N/A	53
Mo_2C/$Ti_3C_2T_x$@NC	N/A	40	6	65	54
PtO_aPdO_b/$Ti_3C_2T_x$	0.14	39	26.5	N/A	55
Pd/Nb_2C	4	43	34	N/A	56
CoMoC/Ti_3C_2-NC	0.2	24	81	99	57
